# Multicystic Interstitial Lung Disease Due to a Novel Biallelic C‐C Chemokine Receptor Type 2 Variant

**DOI:** 10.1002/ppul.71135

**Published:** 2025-05-27

**Authors:** Moritz Herkner, Christina Rapp, Simon Y. Graeber, Charlotte Marx, Carlotta Rambuscheck, Simone Reu‐Hofer, Nagehan Emiralioglu, Nural Kiper, Alexandru I. Gilea, Ilenia Notaroberto, Enrico Baruffini, Bettina Temmesfeld‐Wollbrück, Christoph Klein, Han Wen, Mirjam Stahl, Matthias Griese, Florian Gothe

**Affiliations:** ^1^ Department of Pediatrics, Dr. von Hauner Children's Hospital, University Hospital Ludwig‐Maximilians‐Universität Munich Munich Germany; ^2^ Department of Pediatric Respiratory Medicine, Immunology and Intensive Care Medicine Charité ‐ Universitätsmedizin Berlin Berlin Germany; ^3^ German Center for Lung Research (DZL) Associated Partner Site Berlin Germany; ^4^ Division of Clinical Pharmacology University Hospital, LMU Munich Munich Germany; ^5^ Institute of Pathology University of Würzburg Würzburg Germany; ^6^ Department of Pediatrics, Division of Pulmonology Hacettepe University Faculty of Medicine Ankara Turkey; ^7^ Department of Chemistry, Life Sciences and Environmental Sustainability University of Parma Parma Italy; ^8^ Department of Infectious Diseases and Pulmonary Medicine Charité ‐ Universitätsmedizin Berlin Berlin Germany; ^9^ Berlin Institute of Health (BIH) at Charité – Universitätsmedizin Berlin Berlin Germany; ^10^ Biochemistry and Biophysics Center, National Heart, Lung, and Blood Institute National Institutes of Health Bethesda Maryland USA; ^11^ German Center for Lung Research (DZL) Comprehensive Pneumology Center Munich Munich Germany

**Keywords:** C‐C chemokine receptor type 2, cystic lung disease, inborn error of immunity, interstitial lung disease

## Abstract

**Objective:**

We are presenting two individuals with biallelic C‐C chemokine receptor type 2 (CCR2) deficiency carrying the novel c.644C>T p.L215P variant, who presented with chronic respiratory symptoms during infancy and developed multiple diffuse cystic lesions during childhood.

**Methods:**

The patients were diagnosed by means of whole exome sequencing and functional validation of the variant was performed in primary patient cells.

**Results:**

While size and extent of the cysts were stable over years, progressive lung function decline was noted in adolescence and adulthood respectively. The CCR2 p.L215P variant was found to be loss‐of‐expression and patient monocytes displayed a migration defect upon stimulation with the CCR2 ligand C‐C motif ligand 2 (CCL2).

**Conclusion:**

With a follow‐up of up to 25 years, this report expands our understanding of lung disease in CCR2 deficiency and offers another monogenic cause of cystic lung disease. Early genetic diagnosis of affected individuals might allow potentially curative treatment by haematopoietic stem cell transplantation.

**Abbreviations**
AM: alveolar macrophages
CCL: C‐C motif ligand
CCR2: C‐C chemokine receptor type 2
COPD: chronic obstructive pulmonary disease
DIP: desquamative interstitial pneumonia
FLCN: folliculin
GM‐CSF: granulocyte‐macrophage colony‐stimulating factor
HRCT: high‐resolution computed tomography
ILD: interstitial lung disease
LAM: lymphangioleiomyomatosis
MAPCA: major aortopulmonary collateral artery
MAPK: mitogen‐activated protein kinase
mTOR: mammalian target of rapamycin
OAS1: oligoadenylatsynthetase 1
PBMC: peripheral blood mononuclear cell
pLCH: pulmonary langerhans cell histiocytosis
TSC: tuberous sclerosis complex

## 1 Introduction

Multiple thin‐walled, well‐defined and circumscribed air‐containing lesions of 1 cm or more in diameter with epithelial or fibrous wall characterize diffuse cystic lung diseases [1]. In recent years, monogenic causes of cystic lung disease have been successively deciphered [2]: Lymphangioleiomyomatosis (LAM) is associated with variants in *tuberous sclerosis complex (TSC) 1* and *TSC2* genes and sporadic LAMs often display somatic mosaicism [3]. Pulmonary Langerhans cell histiocytosis (pLCH) is driven by activating variants in the mitogen‐activated protein kinase (MAPK) pathway [4], and variants in *Folliculin (FLCN)* cause Birt‐Hogg‐Dubé syndrome [5]. Very recently, the first individuals harboring biallelic variants in *C‐C motif chemokine receptor type 2 (CCR2)* have been found to suffer from progressive polycystic lung disease [6]. CCR2 is expressed on monocytes and other myeloid cell populations and its interaction partner C‐C motif ligand 2 (CCL2) acts as a potent chemoattractant guiding monocyte migration into tissues.

Here, we are reporting two cases from a large consanguineous kindred displaying diffuse lung cysts and progressive respiratory decline harboring a new loss‐of‐function variant in *CCR2*.

## 2 Case Reports

The index patient (P1) came to medical attention at the age of 4 months with cough and fever.

He had received a BCG vaccination postnatally without local or systemic complications, but the positive tuberculin skin test result prompted a chest X‐ray examination, which revealed bilateral interstitial infiltrates. The patient was treated for suspected infections with *Mycobacterium tuberculosis*, *Cytomegalovirus*, or *Pneumocystis jirovecii*, but none of these pathogens could be detected in bronchoalveolar lavage fluid (BAL). Surfactant proteins were found present, but no information on BAL cytology is available. The treating physicians also noted to have ruled out primary or secondary immunodeficiency at the time.

**Figure 1:**
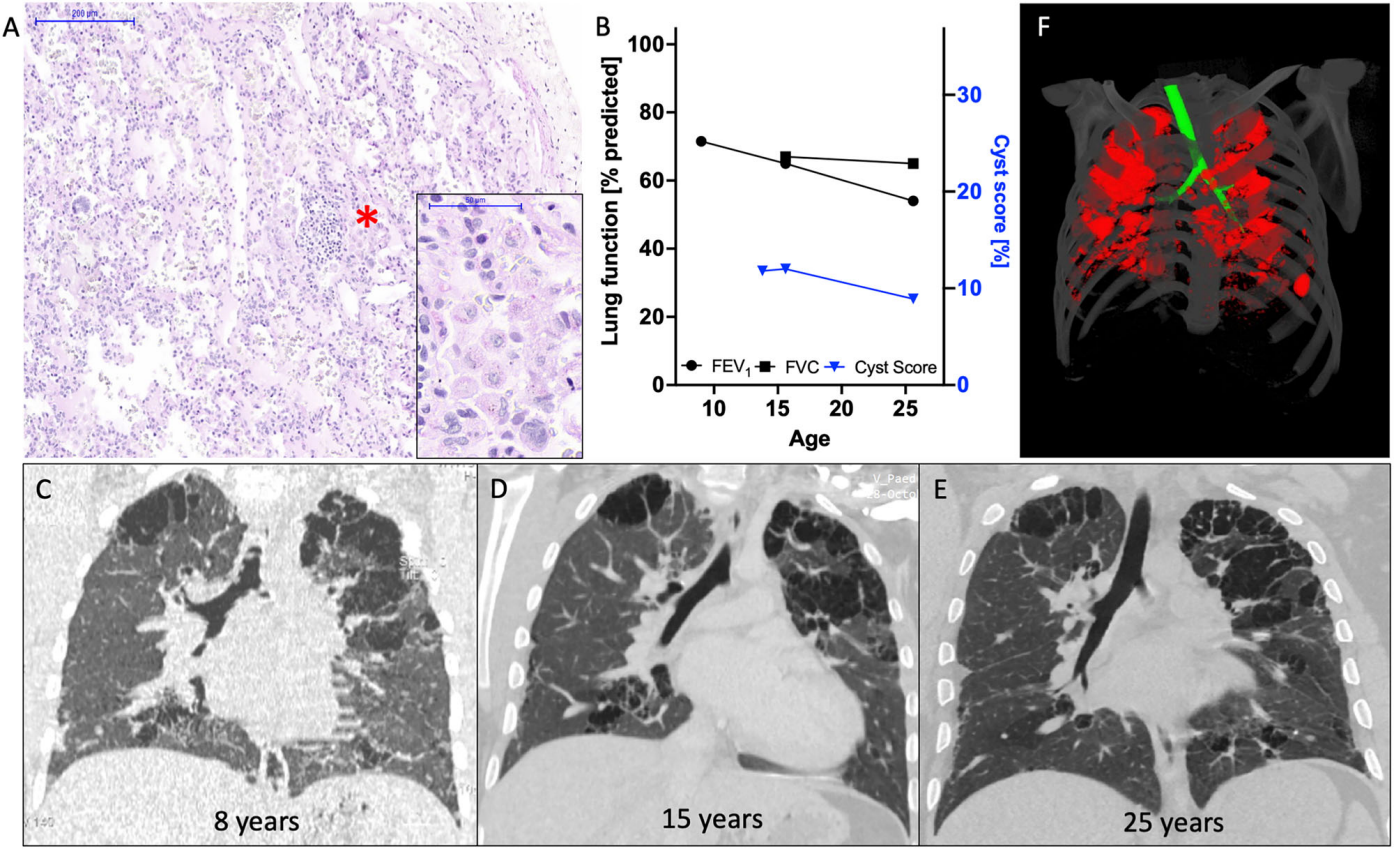
Lung disease in patient 1. (A) Desquamative interstitial pneumonia with intraluminal macrophage accumulation (*, enlarged window) in P1, at the age of 8 months (H&E stain). (B) Pulmonary function tests in P1 with. FEV1 (% predicted, black dots) and FVC (% predicted, black squares) as well as cyst scores (% of total lung volume, blue triangles). (C–E) Coronar high‐resolution chest CT scans of P1 at the age of 8 (C), 15 (D) and 25 (E) years showing radiologically stable, multicystic lung disease. (F) Representative image of cyst extent (red) in P1 at age 25. Large airways are depicted in green. [Color figure can be viewed at wileyonlinelibrary.com]

A lung biopsy performed at the age of 10 months showed an accumulation of alveolar macrophages within the alveoli consistent with desquamative interstitial pneumonia (DIP) (Figure 1A), cholesterol clefts and discrete pleural fibrosis. Notably, no granulocytic reaction was seen. The local pathologists evaluated another BAL at the age of 4 years and there was no evidence at that time of extracellular debris and pulmonary alveolar proteinosis. Repeated chest HRCT scans over 17 years revealed multiple clustered cystic lesions with variable size, predominant in lung apices, perihilar, subpleural, and peribronchial (Figure 1C–E). While peripheral arterial oxygen saturation and 12‐min walking tests were found normal during his first years of life, a restrictive airflow limitation was noted at the age of 10, which worsened during adolescence and early adulthood (Figure 1B). Pulmonary hypertension was excluded by echocardiography.

Although digital clubbing became apparent already in late infancy, the patient reported good exercise tolerance and did not suffer from major infections during childhood. As an adult, exercise intolerance became apparent (Figure 1B). The latest immunological evaluation revealed only a reduced IgG4 subclass level. The patient is currently in a stable clinical condition without regular medication.

P2 suffered from recurrent febrile respiratory tract infections since the age of 5 months. He developed failure to thrive and a chronic wet cough. At the age of 4 years, major aortopulmonary collateral arteries (MAPCAs) were coiled, a procedure repeated when he was 10 years. Clubbing was noted at the age of 5 years but not further investigated.

At age 10, the patient attended respiratory medicine specialist care. He displayed eosinophilia (10%) in full blood count analysis with normal serum IgE levels. However, IgG and IgA were elevated with normal IgM. Lymphocyte subpopulations were grossly normal, only a slight decrease in the naïve CD4^+^ T cell subpopulation with corresponding expansion of the effector memory CD4^+^ T cell compartment was observed. Repeated nitroblue tetrazolium tests ruled out granulocyte dysfunction. He showed normal alpha_1_‐antitrypsin levels, negative ANAs and ANCAs, and a negative Quantiferon test. However, elevated IgG antibodies against pigeon antigen were found.

**Figure 2:**
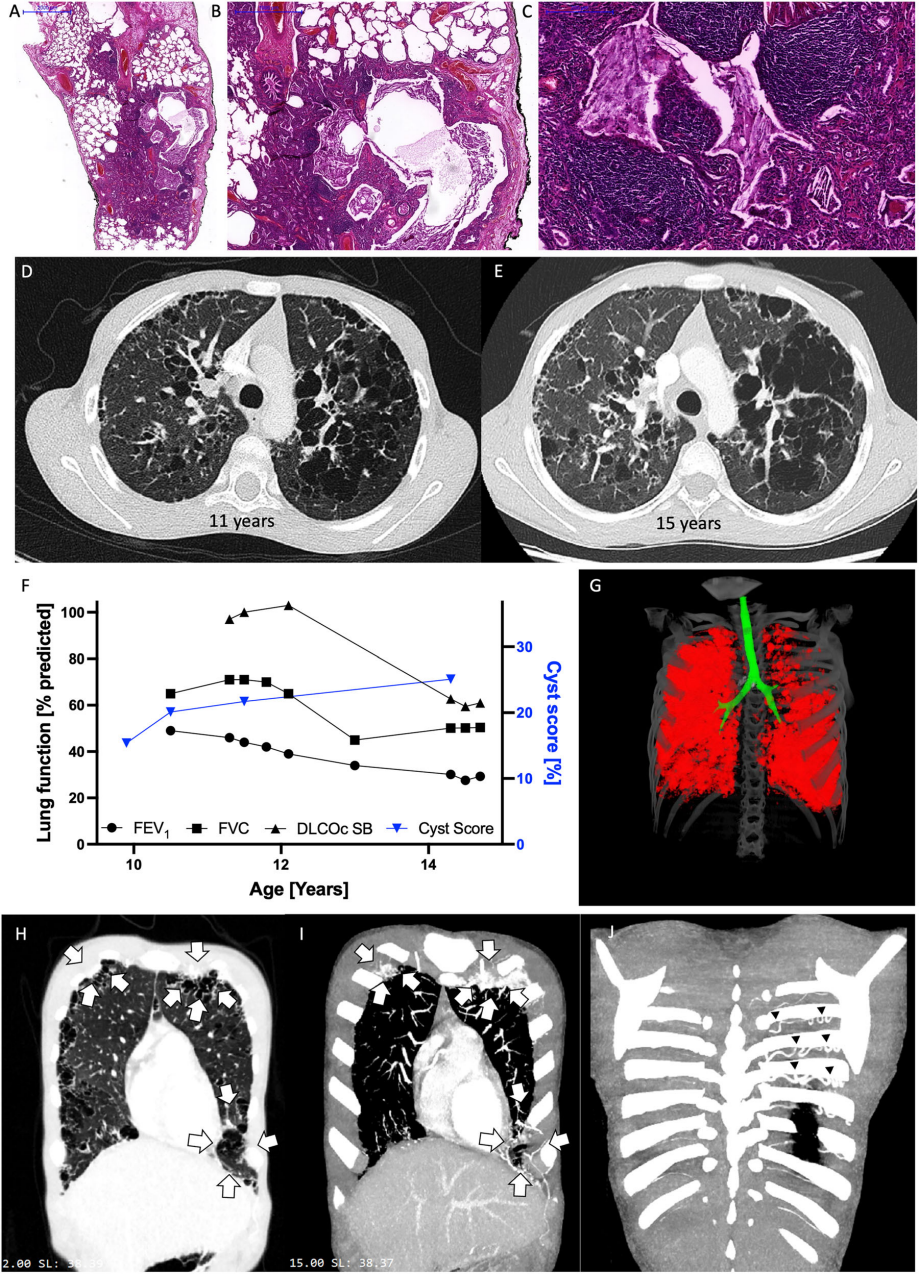
Disease evolution in patient 2. (A–C) Lung biopsy of P2 at the age of 11 years showing multiple small cysts, widened airspaces and intraluminal eosinophilic material with cholesterol clefts at varying magnifications (Figure 2B shows part of Figure 2A at higher magnification), see scale bars (H&E stain). (D and E) Axial chest HRCT scans of P2 at the age of 11 (D) and 15 years (E). (F) Lung function in P2 over time with FEV_1_ (% predicted, black dots), FVC (% predicted, black squares), DLCOc SB (% predicted, black triangle) and cyst scoring (% of total lung volume, blue triangle). (G) Representative image displaying cyst distribution (red) and large airways (green) in P2 at the age of 11. (H) 2 mm coronary section showing cystically destroyed lung tissue (arrows), (I) 15 mm MIP reconstruction with intense vascularization in these areas (arrows) on the same section level. (J) Dorsal section with dilated intercostal arteries increasing aorto‐pulmonary blood supply (arrow heads). [Color figure can be viewed at wileyonlinelibrary.com]

To uncover the cause of his chronic respiratory complaints, an open lung biopsy was performed. While the local pathologists suggested hypersensitivity pneumonitis, reference pathology noted patchy areas of cholesterol pneumonia with cystically dilated airways. Additionally, a chronic interstitial inflammatory infiltrate with prominent lymphofollicular hyperplasia and intraluminal cholesterol clefts was seen (Figure 2A–C).

HRCT showed multicystic lung destruction with centrilobular emphysema (Figure 2D,E).

The patient's lung function showed progressive obstructive flow limitation (Figure 1F) and a reduced diffusion capacity at the age of 15.

Based on the antipigeon IgG antibodies and the initial pathology evaluation, the diagnosis of hypersensitivity pneumonitis was made and steroid pulses were commenced alongside hydroxychloroquine treatment without major benefit. Additional inhaled combination therapy (Beclometason/Formoterol/Glycopyrronium) lead to clinical improvement. The most recent evaluation showed iron deficiency anaemia and normal lymphocyte populations with only a slight reduction of class‐switched memory B cells. IgG and IgA levels were still above the normal range suggesting chronic inflammation. IgE, IgM, as well as IgG subclasses were normal with specific antibodies to tetanus toxoid being present. Weakly elevated IgG responses to *Aspergillus fumigatus*, *Cladosporium herbarum*, *Alternaria alternata*, pigeon, and budgerigar antigens were found. The latest HRCT revealed the presence of additional aortopulmonary collaterals (Figure 2H–J) with echocardiography showing mild left ventricular hypertrophy without the need for another interventional closure procedure. Right ventricular systolic pressure was estimated 32 mmHg via tricuspid insufficiency. At age 16, the patient presented with clubbing, kachexia, and exercise dyspnoea and is currently undergoing evaluation for lung transplantation.

## 3 Methods

### 3.1 Human Subjects

Informed consent was provided by all human subjects or their legal guardians in accordance with the 1975 Helsinki principles for enrollment in research protocols that were approved by the Institutional Review Board of the LMU Munich (EK111‐13). Patients were followed in the chILD‐EU registry (www.childeu.net).

### 3.2 Exome Sequence Analysis

Whole Exome Sequencing (WES) was performed at the Dr. von Hauner Children's Hospital next generation sequencing facility. Genomic DNA was isolated from whole blood (Qiagen) for generation of whole exome libraries using the SureSelect XT Human All Exon V6+UTR kit (Agilent Technologies). Barcoded libraries were sequenced with a NextSeq. 500 platform (Illumina) to an average coverage depth of 90x. Bioinformatics analysis used Burrows‐Wheeler Aligner (BWA 0.7.15), Genome Analysis ToolKit (GATK 3.6) 2 and Variant Effect Predictor (VEP89) 3. The frequency filtering used allele frequencies from the public gnomAD database and a comprehensive in‐house database. The sequence variant and its segregation was confirmed by Sanger sequencing.

### 3.3 Protein Expression

EDTA‐blood from P2, his parents and a travel control (healthy adult volunteer) were stained with CD11c (BV421, Cat. 301627), CD14 (BV786, Cat. 301839), CD16 (BV711, Cat. 302043), CD24 (APC‐Cy7, Cat. 311131), CD45 (AF700, Cat. 304023), CD123 (BV605, Cart. 306025), HLA‐DR (PE‐Cy7, Cat. 327017), and CCR2 (PE, Cat. 357205, all from Biolegend) and gated according to Yu et al. [7]. The gating strategy is displayed in Figure E1. Red cells were lysed using BS FACS Lysing solution and cells were washed twice with FACS buffer before analyzed on a BD LSRFortessa X‐20(BD Bioscience) cytometer. Data were analyzed using FlowJo Software (TreeStar, v10). A fluorescence minus one control was used to determine nonspecific staining detected in the PE channel.

### 3.4 Transwell Migration Assay

Frozen healthy and patient peripheral blood mononuclear cells (PBMCs) from P2 were thawed in RPMI containing 10% FCS and incubated for 2 h at 37°C. After recovering, cells were resuspended in RPMI at a density of 1 × 10^7^/mL.

1 × 10^6^ PBMCs were placed in the upper transwell chamber (Corning, CLS3388) while the bottom of the wells were filled with 235 µl of RPMI containing CCL2 (PeproTech, 300‐04,) or CCL5 (PeproTech, 300‐06) as chemoattractants at a concentration of 60 ng/mL, respectively. The cells were allowed to migrate for 4 h at 37°C and 5% CO_2_ in the incubator. Migrated cells in the bottom compartment were subsequently stained for CD14 (BV605, Cat. 564055, BD) and a viability dye (Invitrogen, Cat. L34994) for 30 min at room temperature in the dark. After staining, cells were fixed with paraformaldehyde 4.2% for 15 min and analysed on a CytoFLEX LX Flow cytometer (Beckman Coulter) with a defined sample volume of 75 uL. Samples were run in duplicates and data represent three independent experiments. Data was analysed using using FlowJo Software (TreeStar, v10).

### 3.5 Automated Cyst Measurement

The automatic method for cyst segmentation utilizes advanced adaptive algorithms to identify and measure pulmonary cysts from volumetric chest CT images [8]. This method is based on local radiodensity thresholds which are derived from the images themselves, effectively removing the need for manual adjustments by operators and thus eliminating human bias. This automated approach is designed to improve consistency over time and across patients when compared with current standard operator‐guided methods. It has been validated in a clinical study involving 152 adult patients with the cystic lung disease lymphangioleiomyomatosis [9].

## 4 Results

Genetics: The parents of both cases originate from the same village in southeastern Turkey. While the parents of P1 are first‐degree cousins, the mother of P2 was also found to be a first‐degree cousin of both parents of P1. It was not possible to pinpoint the suspected blood bond of P2's father, who is also carrying the very rare *CCR2* p.L215P allele in heterozygous fashion, to the other three parents indicating distant consanguinity.

### 4.1 Functional Validation of the Novel Variant

**Figure 3:**
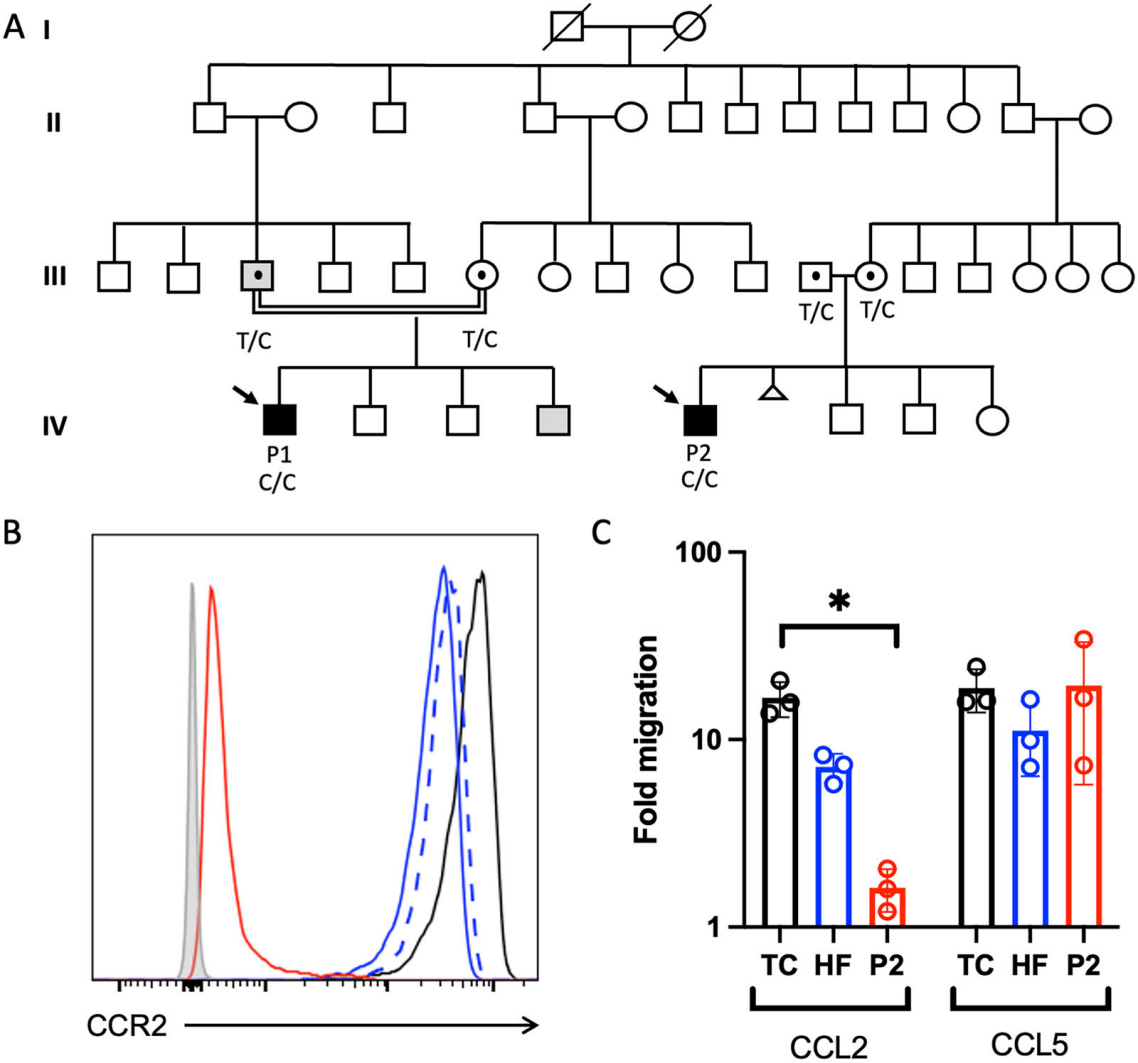
Segregation and functional validation of the *CCR2* p.L215P variant. (A) Pedigree of the large consanguineous kindred. Gray filling indicates diagnosis of asthma in the father and youngest brother of P1. Presence of the p.L215P variant in heterozygous fashion in the parents of both patients is shown by the black dot. (B) CCR2 expression on primary CD14+CD16‐ monocytes in CCR2 deficiency. Travel control (black line), heterozygous mother (dashed blue line) heterozygous father (blue line), P2 (red line), and fluorescence minus one‐control (gray filling), *n* = 3. (C) Transwell migration assay of primary CD14+ monocytes stimulated with CCL2 or CCL5 (60 ng/mL each) for 4 h at 37°C. Travel control (TC, black line), heterozygous father (HF, blue line), P2 (P2, red line). Results display mean and standard deviation of three independent experiments. [Color figure can be viewed at wileyonlinelibrary.com]

Whole exome sequencing revealed a homozygous missense variant in *CCR2*, c.644C>T, p.L215P in both patients, absent from public databases and segregating with disease (Figure 3A).

To investigate a potential functional impact of the amino acid exchange, we evaluated expression of the mutated CCR2 protein in peripheral blood mononuclear cells of P2 and his heterozygous parents (Figure 3B). Classical monocytes (CD14^+^, CD16^−^) of the patient exhibited 1% of CCR2 levels seen in the healthy travel control, cells of the heterozygous parents showed a 50% reduction. A similar loss‐of‐expression pattern was also detected in nonclassical monocytes (CD14^+^, CD16^intermediate/high^), basophils, and plasmacytoid dendritic cell populations of P2. To finally prove defective CCR2 function, we employed a migration assay using CCL2, as well as CCL5 as a positive control (Figure 3C). Whereas stimulation with CCL5 induced comparable migration through a transwell in primary monocytes of P2, his father, and healthy control cells, CCL2 failed to promote monocyte migration in patient cells.

### 4.2 Cyst Evolvement Over Time

Using an automated cyst scoring method, we were able to quantify cyst volume in both patients over time (Figures 1F and 2G). While the fist CT scan in P1 performed at the age of 8 could not be assessed via this method due to quality limitations, we observed stable cyst size of 9‐12% of total lung volume from age 14 onwards (Figure 1B). In P2, an increase of the cyst score (total cyst volume/total lung volume) corresponding to the observed lung function decline was noted (Figure 2F). However, cyst scoring in the first CT scan of P2 at age 9 was complicated by excessive image blurring due to patient movement possibly leading to underestimation of cyst volumes in this particular scan.

## 5 Discussion

We are presenting two cases of autosomal‐recessive CCR2 deficiency suffering from interstitial lung disease (ILD) presenting with cystic lung destruction. Chronic respiratory symptoms and clubbing were noted during the first years of life. Radiologically, lung cysts developed early in childhood, show a bilateral diffuse distribution pronounced in the upper lobes and were stable (P1) or slowly progressing (P2) in size and extent over many years up into adulthood. It is interesting to note that lung biopsy in both patients revealed cholesterol clefts, pointing towards a macrophage dysfunction. The presence of alveolar macrophages (AM) in the lungs was not affected by the lack of CCR2, they even accumulate in the alveolar lumen appearing as desquamative interstitial pneumonia in P1. P2, on the other hand, presented a marked lymphocytic infiltrate.

These case studies parallel the ones presented by Neehus and coworkers in many respects: First, the *CCR2* variants all lead to a loss of CCR2 expression. Some patients suffered from recurrent respiratory infections during childhood like P2 and clubbing was noted as an early disease manifestation in 4/6 patients [6]. Lung biopsy findings are discussed in 2/9 patients and showed cholesterol clefts and lymphocyte accumulation like in P2 [6]. In contrast to the patients reported by Neehus et al. we did not observe extracellular debris in the alveolar space and evidence of pulmonary alveolar proteinosis. Unfortunately, we have no information on macroscopic appearance of the BAL fluid or its cellular composition in our cases. Most, but not all individuals in the Neehus study displayed multiple lung cysts with upper lobe prominence like in our cases. An increase in cyst size and number was noted in 2/3 patients where serial CT scans were available [6]. Lung function testing usually revealed progressive obstructive airflow limitation as seen in P2.

CCR2 is expressed on myeloid cell populations and interaction with different ligands guides monocytes into sites of inflammation. It is important to understand that AM originate from fetal progenitors, populate the lung tissue perinatally and self‐maintain their population through low‐grade proliferation, processes all independent of CCR2 [10]. Only following lung infection or other inflammatory insults, AM are replenished by invading Ccr2^+^ bone marrow–derived monocytes [11], a process abrogated in *Ccr2‐*deficient mice [12]. In CCR2 deficiency, this defective monocyte recruitment to the lung is likely responsible for the observed susceptibility to recurrent airway infections and BCG complications. It is tempting to speculate that accumulation of different immune cell populations reflects an attempt to compensate for defective monocyte recruitment in CCR2‐deficient individuals. In accordance, cystic lung disease has also been observed in other lymphoproliferative lung diseases like lymphoid interstitial pneumonia of unknown aetiology [13].

CCR2 has also been implicated in more frequent lung diseases of adulthood: Ccr2^+^ cells accumulate in experimental murine pulmonary fibrosis [14, 15] and have also been found enriched in human idiopathic pulmonary fibrosis [16]. The CCR2 ligand CCL2, produced by bronchial epithelial cells, is increased in the sputum of chronic obstructive pulmonary disease (COPD) patients [17] and recent data in CCL2 knock‐out mice supports an important role of the CCL2‐CCR2 axis in the development of lung emphysema [18].

Unexpectedly, we found normal (P2) or even increased (P1) macrophage numbers in our biopsies whereas Neehus et al. reported reduced macrophage content in BAL fluid and lung biopsy samples [6]. Only possibly explanation is that the AM pool at the age of 10 months, when lung biopsy was performed in P1, might still have been large enough to allow for their accumulation in the alveolar space. The other three biospies in CCR2 deficient individuals, where detailed information is available (two by Neehus and colleagues), were taken at later timepoints (P1 Neehus 8 years; P7 Neehus 3 years; P2 11 years, respectively).

Given the current small number of CCR2 deficient individuals it is likely that the spectrum of CCR2 deficiency disease will be expanded in the future as more cases are diagnosed. Unfortunately, our case descriptions also contain missing data due to the retrospective collection. Information on the MAPCAs in P2 for example, is sparse and it is therefore impossible to understand whether they are related to CCR2 deficiency or a coincident finding. However, it is very likely that these dilated arteries are related to chronic lung disease as repetitive interventions at 4 and 10 years of age were necessary and on the latest CT scan again large intercostal vessels originating from the aorta are clearly visible.

It is surprising that a global defect of monocyte recruitment to tissues seems to result in an almost exclusive pulmonary phenotype. However, a similar picture is seen in granulocyte‐macrophage colony‐stimulating factor (GM‐CSF) receptor deficiencies, where a cytokine signaling defect supposedly crucial to support survival of tissue resident macrophages throughout the body also leads to a lung‐only disease. One might hypothesize haematopoietic stem cell transplantation offering a specific treatment option for CCR2‐deficient individuals as this procedure has been shown to cure other monocyte defects with a dominant pulmonary phenotype like GATA2 haploinsufficiency [19], *Oligoadenylatsynthetase 1 (OAS1)*‐related disease [20], or interferon regulatory factor 8 deficiency [21, 22]. However, since cysts appeared within the first 10 years of life, a timely genetic diagnosis is necessary. This highlights the importance of early genetic testing in ILD cases, since specific treatments are becoming more and more available. This holds true also for other cystic lung diseases like mTOR inhibition in TSC‐related LAM [3] or Vemurafenib in pLCH [4]. Taken together, CCR2 deficiency constitutes a new inborn error of pulmonary immunity leading to progressive cystic interstitial lung disease.

### 5.1 Clinical Implications

Biallelic CCR2 deficiency displays exclusive pulmonary involvement. Multicystic lung destruction starts early in childhood. A timely genetic diagnosis is essential to potentially refer patients to curative haematopoietic stem cell transplantation.

## Author Contributions

**Moritz Herkner:** investigation, methodology, visualization, writing – review and editing. **Christina Rapp:** investigation, writing – review and editing, methodology. **Simon Y. Graeber:** writing – review and editing, methodology. **Charlotte Marx:** methodology, writing – review and editing. **Carlotta Rambuscheck:** methodology, writing – review and editing. **Simone Reu‐Hofer:** visualization, methodology, writing – review and editing. **Nagehan Emiralioglu:** methodology, writing – review and editing. **Nural Kiper:** methodology, writing – review and editing. **Alexandru I Gilea:** methodology, writing – review and editing. **Ilenia Notaroberto:** methodology, writing – review and editing. **Enrico Baruffini:** methodology, writing – review and editing. **Bettina Temmesfeld‐Wollbrück:** methodology, writing – review and editing. **Christoph Klein:** methodology, writing – review and editing. **Han Wen:** methodology, software, visualization, writing – review and editing. **Mirjam Stahl:** methodology, writing – review and editing, supervision. **Matthias Griese:** methodology, conceptualization, investigation, funding acquisition, supervision. **Florian Gothe:** supervision, writing – review and editing, writing – original draft, methodology, conceptualization, investigation, funding acquisition.

## Conflicts of Interest

The authors declare no conflicts of interest.

## Supporting information

Fig E1: Gating strategy to identify the indicated myeloid cell population in whole blood and their respective CCR2 expression. FMO: fluorescence minus one control; pDCs: plasmacytoid dentritic cells.

## Data Availability

The data that support the findings of this study are available from the corresponding author upon reasonable request.
