# Supplementary figures and images for: Multicystic Interstitial Lung Disease Due to a Novel Biallelic C‐C Chemokine Receptor Type 2 Variant
Source: Pediatr Pulmonol. 2025 May 27;60(5):e71135. doi: 10.1002/ppul.71135 (PMC12117283; doi:10.1002/ppul.71135)

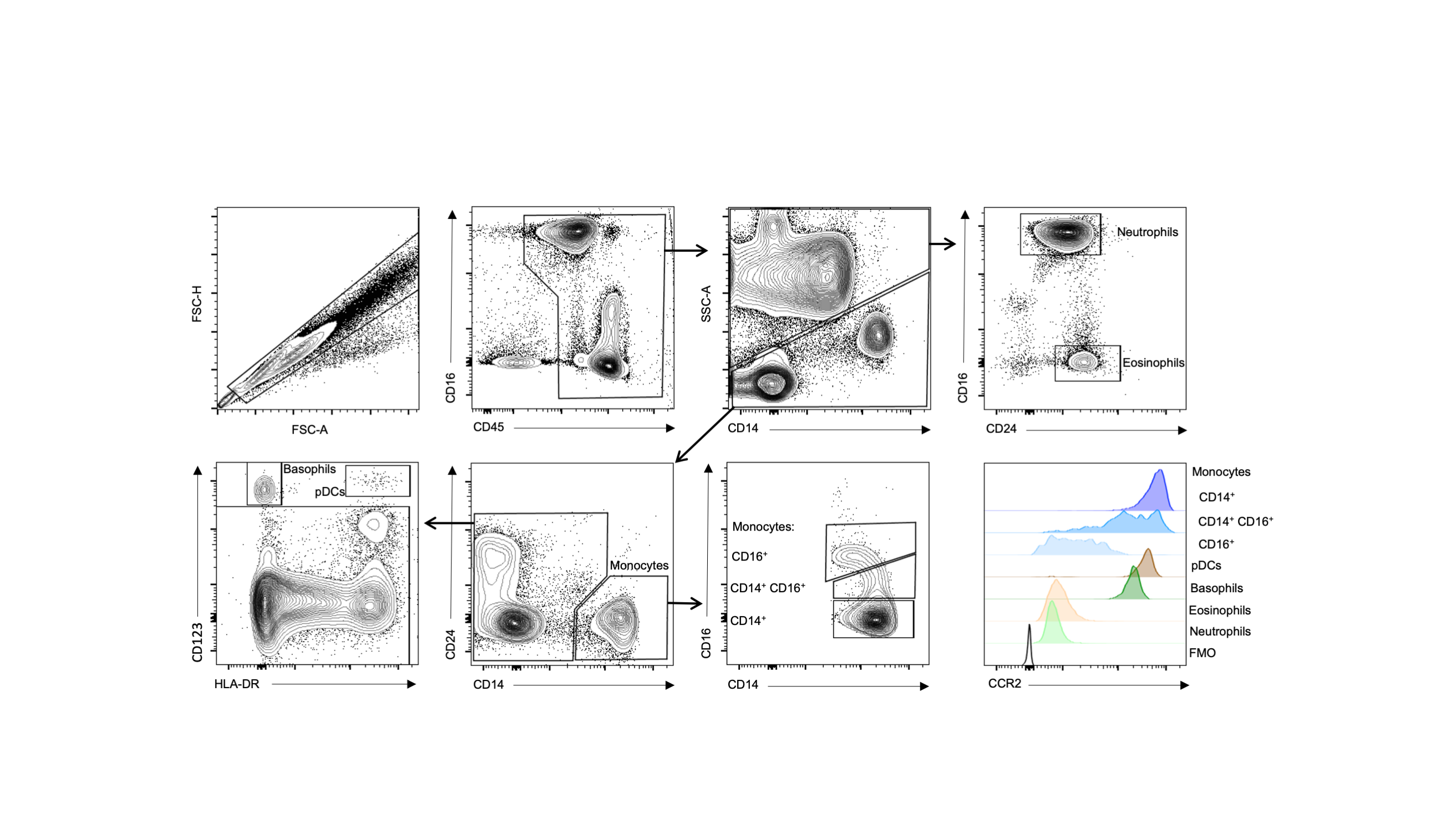

Supplement: Supplementary file 1 — Fig E1: Gating strategy to identify the indicated myeloid cell population in whole blood and their respective CCR2 expression. FMO: fluorescence minus one control; pDCs: plasmacytoid dentritic cells. [file PPUL-60-0-s001.tiff]
